# B lymphocytes and B-cell activating factor promote collagen and profibrotic markers expression by dermal fibroblasts in systemic sclerosis

**DOI:** 10.1186/ar4352

**Published:** 2013-10-28

**Authors:** Antoine François, Emmanuel Chatelus, Dominique Wachsmann, Jean Sibilia, Seiamak Bahram, Ghada Alsaleh, Jacques-Eric Gottenberg

**Affiliations:** 1 Immunorhumatologie Moléculaire, INSERM UMR_S 1109, Centre de Recherche en Immunologie et Hématologie, Fédération de Médecine Translationnelle de Strasbourg, Université de Strasbourg, Strasbourg, France; 2 Service de Rhumatologie, Centre National de Référence pour les Maladies Systémiques Autoimmunes Rares, Hôpitaux Universitaires de Strasbourg, Avenue Moliere, 67098 Strasbourg Cedex, France

## Abstract

**Introduction:**

B lymphocytes might play a pathogenic role in dermal fibrosis in systemic sclerosis (SSc). B-cell activating factor (BAFF), a key cytokine for B-cell activation, is increased in the serum and the skin of patients with SSc. However, the ability of B cells directly to stimulate dermal fibroblasts and the role of BAFF are not fully understood. We therefore investigated the involvement of B cells and BAFF in the expression of collagen and profibrotic markers by dermal fibroblasts.

**Methods:**

Cocultures of blood B cells from healthy blood donors and normal or SSc dermal fibroblasts stimulated with anti-IgM and BAFF were performed. *Alpha-SMA*, *TIMP1*, *MMP9*, *COL1A1*, *COL1A2,* and *COL3A1* mRNA expression were determined by quantitative RT-PCR. Soluble collagen, BAFF, IL-6, IL-1β, TGF-β1, and CCL2 protein secretion were assessed.

**Results:**

Coculture of blood B cells and dermal fibroblasts isolated from SSc patients induced IL-6, TGF-β1, CCL2, and collagen secretion, as well as *Alpha-SMA*, *TIMP1,* and *MMP9* expression in dermal fibroblasts. Transwell assays demonstrated that this induction was dependent on cell-cell contact. Addition of anti-IgM and BAFF to the coculture increased IL-6, CCL2, TGF-β1, and collagen secretion. B cell- and BAFF-induced collagen secretion was highly reduced by anti-TGF-β1 antibodies.

**Conclusions:**

Our results showed for the first time a direct role of B cells on the production of collagen by dermal fibroblasts, which is further enhanced by BAFF. Thus, these results demonstrate a new pathogenic role of B cells and BAFF in fibrosis and systemic sclerosis.

## Introduction

Systemic sclerosis (SSc) is a systemic autoimmune disease that has a complex pathogenesis involving genetic and environmental factors [1,2]. SSc is characterized by vascular hyperreactivity, skin and visceral organs fibrosis, and immunologic alterations, including production of autoantibodies [3]. Fibrosis results from excessive collagen production by fibroblasts, and recent studies uncovered that B cells might play a role in the development of fibrosis. It was demonstrated that B cell-deficient mice treated with CC1_4_ to trigger hepatic fibrosis showed a reduced collagen deposition by a mechanism dependent on antibodies but independent of T cells [4]. Likewise, CD19-deficient mice exhibit a reduced susceptibility to pulmonary fibrosis after bleomycin challenge, whereas CD19 overexpression exacerbates fibrosis [5]. SSc patients also have B-cells abnormalities such as the production of specific autoantibodies. Moreover, the presence of CD20^+^ B cells and immunoglobulin genes were detected in skin biopsies of SSc patients [6,7]. B cells are a source of IL-6 and TGF-β1, which have been shown to regulate collagen synthesis by fibroblasts [8]. In SSc patients, IL-6 serum levels correlate with skin fibrosis, and IL-6-deficient mice have attenuated collagen deposition in lungs after bleomycin challenge [9,10]. TGF-β1 also has the ability to inhibit collagen degradation by decreasing matrix metalloproteinases (MMPs) and increasing tissue inhibitor of metalloproteinases (TIMPs) expression [11].

Survival of peripheral B cells is crucially dependent on B cell-activating factor (BAFF) and a proliferation-inducing ligand (APRIL) [12]. The finding that BAFF-transgenic mice develop autoimmune manifestations with similarities to systemic lupus erythematosus and Sjögren syndrome in humans suggested a critical role of BAFF in autoimmune diseases [13,14]. Elevated levels of BAFF have been detected in serum and skin samples from patients with SSc, which suggests that this cytokine contributes to B-cell abnormalities and disease development in patients with SSc [15,16].

The pathogenic role of B cells and BAFF in SSc might not be restricted to secretion of immunoglobulins, antigen presentation, or cytokine secretion. However, to date, no study addressed the ability of B cells to stimulate fibroblasts directly. To investigate the involvement of B cells in dermal fibrosis, we used a coculture model of human dermal fibroblasts (HDFs) isolated from healthy controls or SSc patients with blood B cells and assessed collagen and profibrotic cytokine and markers expression. The present study demonstrates that B cells and BAFF are capable of stimulating collagen secretion by dermal fibroblasts.

## Methods

### Patients and cells

Primary cultures of human dermal fibroblasts (HDFs) were established by outgrowth of cells from explanted tissue pieces. Skin biopsies were obtained by punch biopsies from three healthy subjects (NHDF) and from six patients with SSc (SScHDF) of the Departement de Rhumatologie, Hôpitaux Universitaires de Strasbourg, France. Blood mononuclear cells were isolated from six healthy blood donors. Approval by the ethical committee of the Hopitaux Universitaires de Strasbourg was obtained. Informed consent was obtained from patients and healthy donors.

Diagnosis of SSc was performed according to the revised criteria of the American College of Rheumatology (ACR). All patients were female and had diffuse cutaneous systemic sclerosis and anti-Scl70-positive antibodies. All biopsies were isolated from the forearm of SSc patients. The modified Rodnan skin scores were 29, 8, 0, 25, 28, and 14, respectively. Two patients were treated with oral prednisone (Cortancyl) (5 or 10 mg/day, respectively), and one patient was treated with methotrexate (10 mg/week). Three patients were treated with both oral prednisone and methotrexate. HDFs were used in the experiments between the third and the sixth passages. Blood mononuclear cells were isolated from healthy blood donors by Ficoll-Paque centrifugation, as described in standard protocols. B cells were then selected by negative sorting by using EasySep Human B Cell Enrichment Kit (Stemcell Technologies Grenoble, France). The efficacy of B-cell isolation was determined with FACS analysis by using anti-CD19 antibodies. The yield of isolated B cells was composed of 99% CD19^+^/CD3^-^ B cells, 0.01% of CD19^-^/CD3^+^ T cells, and 0.09% of CD19^-^/CD3^-^ cells.

### Cultures and reagents

Cells were cultured in RPMI1640 supplemented with fetal calf serum (FCS), penicillin, streptomycin, amphotericin B (all from Invitrogen). HDF (10^5^ cells) alone, B cells (3 × 10^5^ or 5 × 10^5^ cells) alone, or cocultures were seeded in 24-well plates for 3 or 5 days. For transwell experiments, B cells (5 × 10^5^ cells) and HDF (10^5^ cells) were seeded in the upper and lower chambers, respectively, of a 0.4-μm polycarbonate membrane transwell (Nunc Dominique Dutscher, Brumath, France). For fibroblasts stimulation, cells were incubated with 5 ng/ml of recombinant TGF-β1 (from R&D Systems Lille, France). For B-cell stimulation, cells were incubated with 100 ng/ml of soluble recombinant human BAFF (rhBAFF; R&D systems Lille, France) in the presence of 5 μg/ml goat F(ab′)_2_ anti-human μ-chain Ab (Jackson ImmunoResearch Laboratories Suffolk, UK) [17]. Anti-LAP-TGF-β1 (AF-246-NA), anti-IL-6 (Clone 6708), anti-integrin α_4_/VLA4/CD49d (Very Late Antigen-4, Clone 2B4), or control IgG (all from R&D Systems Lille, France) were incubated at 20 μg/ml, unless specified in the figure legends.

### Real-time quantitative RT-PCR

Total RNA was extracted from cells by using the phenol/chloroform separation method, as described in standard protocols (Trizol; Invitrogen Fischer Scientific, Illkirch, France) and then reverse-transcribed by using iScript cDNA Synthesis Kit (BioRad Marnes-la-Coquette, France). Real-time quantitative PCR was performed in a total volume of 20 μl by using SensiMix Plus SYBR kit (Quantace; Corbett Life Science Qiagen, Courtaboeuf, France). After an initial incubation at 95°C for 10 minutes, samples were subjected to 40 rounds of amplification for 15 seconds at 95°C, 15 seconds at 60°C, and 25 seconds at 72°C by using a Rotor-Gene 6000 real-time PCR machine (Qiagen Courtaboeuf, France). Melting-curve analysis was performed to assess the specificity of PCR products. The fold of a specific gene was calculated according to the equation

$$\text{fold}=2^{‒\left(\Delta^{\mathrm{Ct}2‒}\Delta^{\mathrm{Ct}1}\right)},$$

in which ΔCt (change in cycle threshold) is the cycle threshold of the test gene minus the cycle threshold of *GAPDH*, Ct2 is a specific sample, and Ct1 is the control sample. The primers used are *GAPDH* forward (5′- GGTGAAGGTCGGAGTCAACGGA-3′) and reverse (5′-GAGGGATCTCGCTCCTGGAAGA-3′); *COL1A1* forward (5′-CACACGTCTCGGTCATGGTA-3′) and reverse (5′-CGGCTCCTGCTCCTCTTAG-3′); *COL1A2* forward (5′-AGCAGGTCCTTGGAAACCTT-3′) and reverse (5′-GAAAAGGAGTTGGACTTGGC-3′); *COL3A1* forward (5′-ATATTTGGCATGGTTCTGGC-3′) and reverse (5′-TGGCTACTTCTCGCTCT GCT-3′); *ACTA2* (α-SMA) forward (5′-GATGGCCACTGCCGCATCCT-3′) and reverse (5′-ACAGGGTCTCTGGGCAGCGG-3′); *MMP9* forward (5′-TTGGTCCACCTGGTTCAACT-3′) and reverse (5′-ACGACGTCTTCCAGTACCGA-3′); *TIMP1* forward (5′-TTGACTTCTGGTGTCCCCAC-3′) and reverse (5′-CTGTTGTTGCTGTGGCTGAT-3′); *BAFF-R (TNFRSF13C)* forward (5′-GATTCCCGGAGACAGAATGA-3′) and reverse (5′-GTGGGTC TGGTGAGCTGG-3′); *TACI (TNFRSF13B)* forward (5′-ATCCCAGTACTGCTCTTCGG-3′) and reverse (5′-CTGAGTAATGAGTGGCCTGG-3′); *BCMA (TNFRSF17)* forward (5′-CAG TCCTGCTCTTTTCCAGG-3′) and reverse (5′-TGGCAGTTTTCGTGCTAATG-3′).

### Enzyme-linked immunosorbent assay

Human BAFF, IL-6, CCL2 (MCP-1), latent and active TGF-β1, and IL-1β were assessed in 3- and 5-day culture supernatants by using a standard ELISA kit according to the manufacturer’s instructions (R&D Systems Lille, France).

### Collagen measurements in cell-culture supernatants

Aliquots of supernatant (200 μl) were assayed for collagen levels and compared with a standard curve prepared from bovine skin by using the Sircol collagen dye-binding assay, according to the manufacturer’s instructions (Biocolor Ltd. Interchim, Montluçon, France).

### B-cell viability assay

B cells alone or cocultured with fibroblasts were seeded in 24-well plates for 3 or 5 days. For transwell experiments, B cells (5 × 10^5^ cells) and HDF (10^5^ cells) were seeded in the upper and lower chambers, respectively, of a 0.4-μm polycarbonate membrane transwell (Nunc). After 3 or 5 days, B cells were stained with 3,3-dihexyloxacarbocyanine iodide (DiOC6) to assess the mitochondrial transmembrane potential (Ψm), and with propidium iodide (PI) to assess membrane permeability. In brief, cell suspensions were incubated with 40 n*M* DiOC6 and 1 μg/ml PI for 15 minutes at 37°C, washed with FACS buffer, and then analyzed on FACS Calibur (BD Biosciences Le Pont de Claix, France). A lymphocyte gate was set by using forward-angle and side-angle light-scatter characteristics of lymphocytes. The vital B cells were brightly positive when stained with DiOC6 and excluded PI.

### Statistical analysis

Values were reported as the median ± interquartile range. Two-tailed Mann–Whitney test was used to compare two independent groups by using GraphPad software. A probability (*P*) value of <0.05 was considered to be significant.

## Results

### B cells induce the production of collagen by dermal fibroblasts from SSc patients and healthy controls

**Figure 1:**
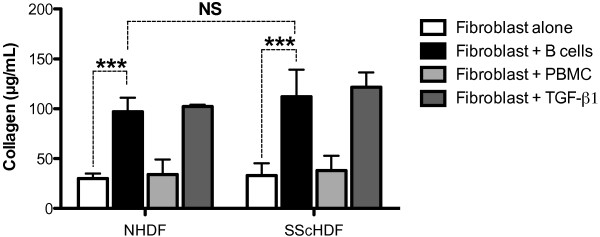
**B cells upregulate collagen secretion by dermal fibroblasts.** Collagen release by dermal fibroblasts was determined by the Sircol collagen dye-binding assay in 3-day culture supernatants of NHDF or SScHDF cultured alone (white bars), cocultured with B cells (black bars), with PBMC (light gray bars), or recombinant TGF-β1 (5 ng/ml, dark gray bars). Data are expressed as the median of duplicate samples from independent experiments with B cells or PBMCs isolated from controls, NHDF from three different healthy individuals, and SScHDF from six different patients with SSc ± interquartile range. NS, not significant; ***P < 0.001.

To study the role of B cells in the induction of collagen synthesis, dermal fibroblasts isolated from patients with SSc (SScHDF) or healthy individuals (NHDF) were cocultured with purified blood B cells isolated from healthy controls. Only 1% of B cells cultured alone were alive at day 5, whereas 49% were alive after coculture with fibroblasts (see Additional file 1: Figure S1). As shown in Figure 1, 5.10^5^ B cells strongly induced collagen secretion by 10^5^ NHDF and SScHDF (black bars), with a magnitude comparable to that of the stimulation with recombinant TGF-β1 (dark gray bars). To confirm that the stimulating effect of B cells was not related to the B cell/fibroblast ratio, additional experiments using 3 × 10^5^ B cells or 5 × 10^5^ B cells were performed and showed that a lower ratio of B cells/fibroblasts was still effective to induce collagen secretion (data not shown). However, no difference was observed between SScHDF or NHDF. Then, to determine whether the induction of collagen in dermal fibroblasts is specific to B cells, we also cocultured dermal fibroblasts with PBMCs. We observed that the levels of collagen in the supernatants were not affected by the presence of PBMCs compared with dermal fibroblasts cultured alone.

### Coculture of B cells and SScHDFs induces the expression of collagen and profibrotic markers

**Figure 2:**
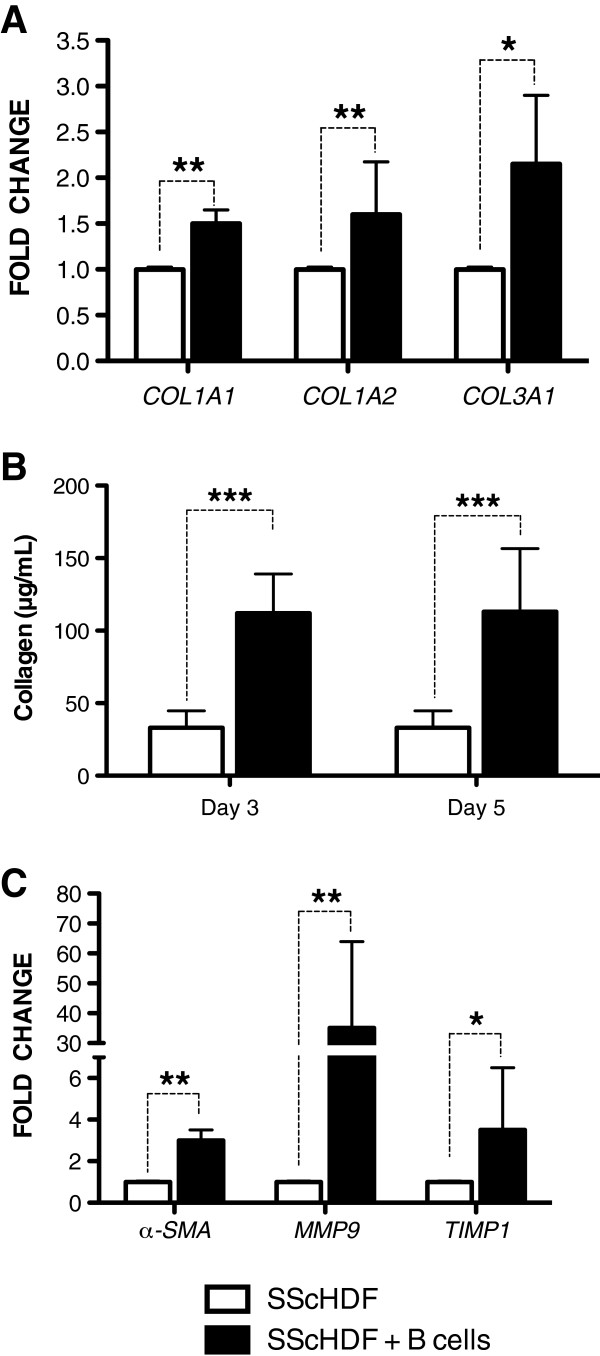
**Expression of collagen and profibrotic markers is increased in SScHDF cocultured with B cells. (A)** Levels of *COL1A1*, *COL1A2,* and *COL3A1* mRNA were determined with RT-qPCR in SScHDF cocultured with blood B cells (black bars) for 3 days. Results were normalized to *GAPDH* and were expressed as the fold change compared with samples from SScHDF alone (white bars). **(B)** Collagen release by SScHDF was determined by the Sircol collagen dye-binding assay in 3- and 5-day culture supernatants. **(C)** Levels of *α-SMA*, *MMP9,* and *TIMP1* mRNA were determined with RT-qPCR in the same conditions as panel A. Data are expressed as the median of duplicate samples of six independent experiments ± interquartile range. *P < 0.05; **P < 0.01; ***P < 0.001.

To study the role of B cells in the expression of collagen and profibrotic markers by SScHDF, we first evaluated mRNA levels of *COL1A1*, *COL1A2,* and *COL3A1*, which encode type I and type III collagens in SScHDF. As shown in Figure 2A, coculture of SScHDF with B cells for 3 days significantly induced the expression of *COL1A1*, *COL1A2,* and *COL3A1*. Moreover, levels of collagen released in the supernatant were significantly higher compared with SScHDF alone after 3 and 5 days of coculture (Figure 2B). Then, we assessed the expression of profibrotic markers with SScHDF. The differentiation of fibroblasts into contractile myofibroblasts expressing α-smooth muscle actin (α-SMA) amplifies the pathologic reparative process in systemic sclerosis. Interestingly, levels of *α-SMA* mRNA were significantly increased in SScHDF when they were cocultured with B cells (Figure 2C). Levels of *TIMP1*, also implicated in tissue remodeling, evolved in a comparable manner to *α-SMA* levels (Figure 2C). Levels of MMP9, known to be increased and well correlated with skin scores in systemic sclerosis [18], were considerably increased in SScHDF cocultured with B cells (Figure 2C). These findings suggest that B cells promote collagen secretion and *α-SMA*, *TIMP1,* and *MMP9* expression by dermal fibroblasts.

### BAFF upregulates the expression of collagen, *α-SMA,* and *TIMP1* in SScHDF cocultured with B cells

**Figure 3:**
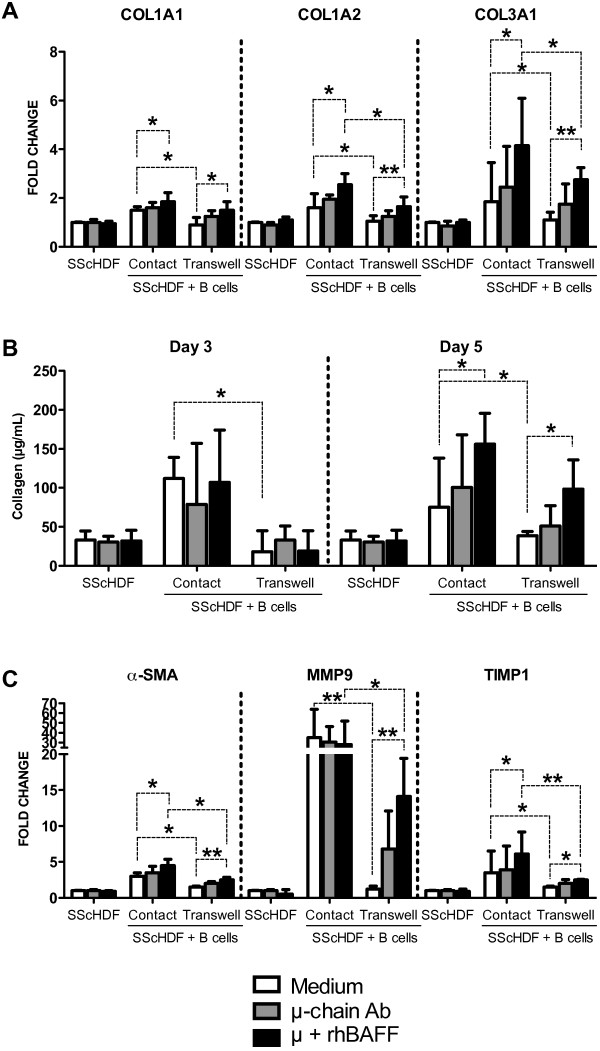
**B cell-induced collagen secretion and profibrotic markers expression are contact dependent, and the role of BAFF is dependent on soluble factors. (A)** Levels of *COL1A1*, *COL1A2,* and *COL3A1* mRNA were determined with RT-qPCR in SScHDF after incubation with medium (white bars), anti-μ-chain antibodies at 5 μg/ml (grey bars), or anti-μ and rhBAFF at 100 ng/ml (black bars) in the presence or absence of blood B cells. B cells were either in contact with SScHDF (contact) or seeded in the upper chamber of a transwell culture insert (transwell). Results were expressed as the fold change compared with samples from SScHDF alone with medium. **(B)** Collagen release by SScHDF was determined by the Sircol collagen dye-binding assay in 3 and 5 days of culture supernatants. **(C)** Levels of *α-SMA*, *MMP9,* and *TIMP1* mRNA were determined with RT-qPCR in the same conditions as in panel **A**. Data are expressed as the median of duplicate samples of six independent experiments ± interquartile range. *P < 0.05; **P < 0.01.

We first determined the level of BAFF in the supernatant of cultured cells. BAFF could not be detected in the supernatant of fibroblasts alone, B cells alone and in the cocultures (data not shown). Because levels of BAFF, secreted mainly by myeloid cells, are importantly increased in SSc, we investigated whether BAFF could amplify collagen secretion. Cocultured B cells and fibroblasts were incubated with anti-human *μ*-chain antibodies, to mimic B cell-receptor engagement, alone or concomitant with soluble BAFF [17]. BAFF did not have any effect on collagen production by SScHDF cultured alone (Figure 3A,B), and no expression by fibroblasts of BCMA, BAFF-R, and TACI could be detected by using qPCR (data not shown). After 3 days of coculture, the addition of BAFF significantly upregulated *COL1A1*, *COL1A2,* and *COL3A1* mRNA levels in SScHDF (Figure 3A). Moreover, a significant upregulation in collagen secretion was detected after 5 days of coculture (Figure 3B). In addition, levels of *α-SMA* and *TIMP1*, but not *MMP9,* mRNA were upregulated in SScHDF by the addition of BAFF in the coculture (Figure 3C).

### Both cell-cell contact and soluble mediators are involved in collagen and profibrotic markers expression

We then evaluated whether the increased collagen secretion induced by B cells is due to soluble or cell-membrane factors. B cell-induced collagen production by SScHDF was completely inhibited by the use of transwells (Figure 3A,B). In addition, transwells completely abrogated the effect of B cells on *α-SMA*, *TIMP-1,* and *MMP9* expression in SScHDF (Figure 3C). Conversely, in the presence of anti-IgM and BAFF, collagen production, as well as *α-SMA*, *TIMP-1,* and *MMP9* expression, were only partially inhibited by transwells (Figure 3A,B,C).

Collectively, these data indicate that B cell-induced collagen secretion by SScHDF is contact dependent, but the effect of BAFF in coculture is, at least partly, dependent on soluble factors.

### Involvement of profibrotic cytokines in B cell- and BAFF-induced fibrosis

**Figure 4:**
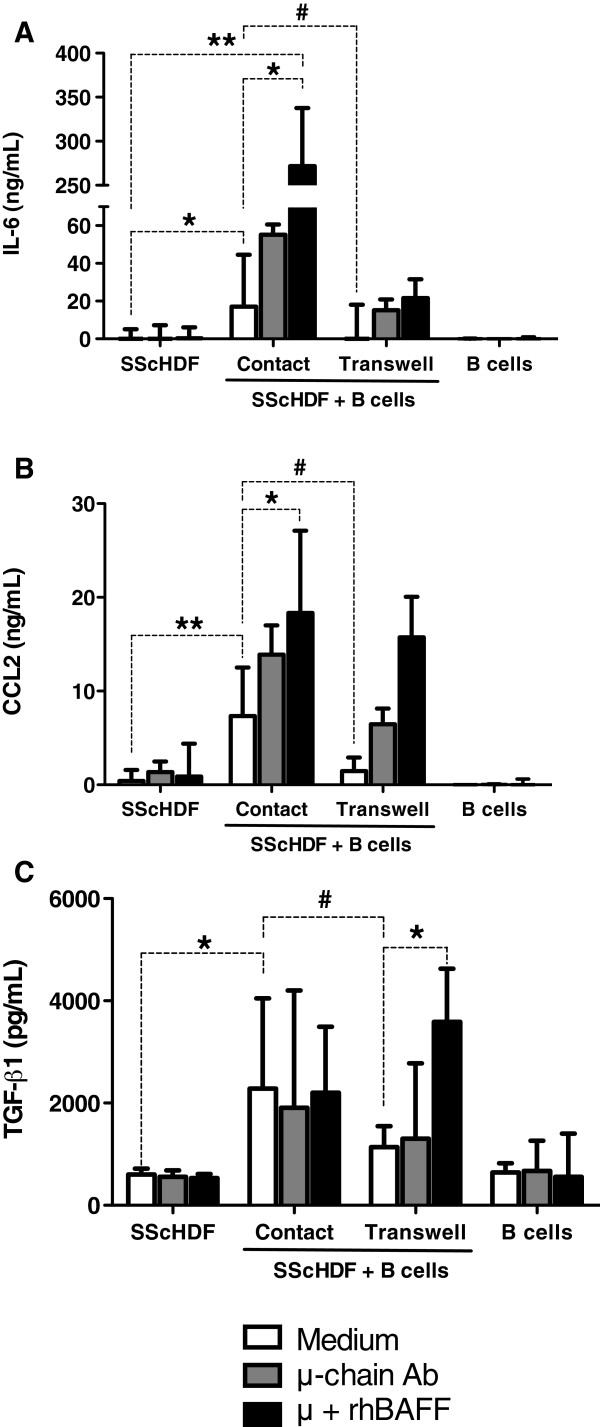
**Profibrotic cytokines and chemokines are upregulated in cocultures. (A**-**C)** Levels of IL-6 **(A)**, C*CL*2 (or MCP-1) **(B)**, and latent TGF-β1 **(C)** were determined by ELISA in the culture supernatants after incubation with medium (white bars), anti-μ-chain antibodies at 5 μg/ml (grey bars), or anti-μ and rhBAFF at 100 ng/ml (black bars) in the presence or absence of blood B cells for 3 days. B cells were either in contact with SScHDF (contact) or seeded in the upper chamber of a transwell culture insert (transwell). Data are expressed as the median of duplicate samples of six independent experiments ± interquartile range. * < 0.05; **P < 0.01; #P < 0.05 for transwell compared with contact.

To investigate the role of soluble factors in the coculture of SScHDF with B cells, we evaluated the levels of IL-6, CCL2, TGF-β1, and IL-1β, cytokines and chemokines that have been shown to contribute to fibrosis. IL-1β was not detected in any condition (data not shown). IL-6 and CCL2 secretion were significantly increased in coculture, and BAFF and anti-IgM enhanced their release (Figure 4A,B). Consistent with these results, inhibition of cell-cell contact by using a transwell abolished IL-6 and CCL2 induction (Figure 4A,B). Active TGF-β1 was not detected in the supernatants of cocultures with or without BAFF. BAFF did not induce the expression of latent TGF-β1 by fibroblasts or B cells cultured alone. Latent TGF-β1 was upregulated by B cells, but BAFF did not increase TGF-β1 secretion (Figure 4C). Interestingly, contact inhibition by transwell did not completely abolish the effect of BAFF stimulation on IL-6 and CCL2 secretion (Figure 4A,B,C). These findings suggest that these cytokines might contribute to the effect of BAFF on fibrosis.

### B cell- and BAFF-induced fibrosis is mediated by TGF-β1

**Figure 5:**
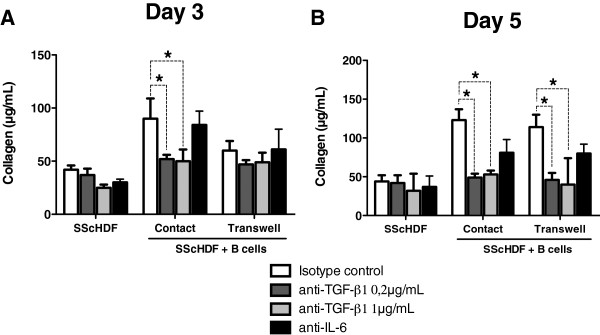
**TGF-β1 is essential for BAFF and B cell-induced collagen upregulation. (A**,**B)** Collagen release by SScHDF was determined by the Sircol collagen dye-binding assay in 3-day **(A)** and 5-day **(B)** culture supernatants after incubation with anti-μ at 5 μg/ml and rhBAFF at 100 ng/ml in all conditions, and isotype control antibody at 20 μg/ml (white bars), anti-TGF-β1 antibodies at 0.2 μg/ml (dark grey bars), anti-TGF-β1 antibodies at 1 μg/ml (light grey bars), or anti-IL-6 antibodies at 20 μg/ml (black bars). Data are expressed as the median of duplicate samples of three independent experiments ± interquartile range. *P < 0.05.

To investigate further the role of soluble factors in B cell- and BAFF-induced collagen release by SScHDF, we used blocking antibodies against the soluble factors that were upregulated in cocultures, IL-6 and TGF-β1. Anti-IL-6 antibodies only slightly reduced the release of collagen by SScHDF in both contact and transwell conditions after 5 days of culture (Figure 5B). Interestingly, blocking TGF-β1 completely abrogated the effect of B cells and BAFF on collagen release by SScHDF, both in contact and transwell conditions (Figure 5A,B).

These data indicate that TGF-β1 plays a pivotal role in the promotion of fibrosis induced by B cells and BAFF.

## Discussion

In this study, we demonstrated that B cells cocultivated with human dermal fibroblasts (HDFs) are potent inducers of collagen and profibrotic cytokine production. As fibrosis results from excessive collagen production by fibroblasts, these data support the hypothesis that B cells could play a role in the development of skin fibrosis in SSc. So far, studies on the role of B cells in SSc focused on their ability to secrete autoantibodies, such as anti-DNA topoisomerase I antibodies that may play a pathogenic role. Hénault *et al.*[19] showed that binding of DNA topoisomerase I to HDF membrane induces monocytes adhesion, in turn activated by anti-topoisomerase I autoantibodies isolated from SSc patients.

Moreover, it is well established that SSc patients show several B-cell abnormalities, such as an overexpression of CD19 as well as a disturbed peripheral homeostasis [20]. SSc patients also show an expansion of naïve B cells and reduced memory B cells and plasmablasts/early plasma cells [21,22]. We investigated the direct interaction between B cells and dermal fibroblasts. First, we demonstrated that B cells increased collagen secretion by dermal fibroblasts with a magnitude comparable to the stimulation of fibroblasts with recombinant TGF-β1. Conversely, PBMCs did not induce collagen secretion, which might be due to the opposite effect of T lymphocytes. It was reported that T lymphocytes inhibit collagen secretion by dermal fibroblasts via membrane-bound TNF-α [23,24]. Interestingly, no difference in terms of collagen secretion was observed between fibroblasts of patients with SSc and healthy individuals. This capacity to increase collagen secretion might also be further enhanced *in vivo* by specific properties of B cells and the local environment observed in the disease. Given that B cells are activated in SSc, it is also possible that SSc B cells could be more-potent inducers of collagen production than normal B cells. This hypothesis could not be tested because the effect of normal B cells on fibroblasts is already so profound that it would be difficult to observe any differences between SSc B cells and normal B cells.

Coculture of B cells with SScHDF induced the secretion of IL-6, CCL2 and TGF-β1, cytokines that are known to stimulate the production of collagen by fibroblasts. Concerning IL-6, our data are consistent with previous observations showing that coculture of a B cell line established from lung tissue of a patient with progressive SSc and normal lung fibroblasts results in the production of high amounts of IL-6 [25]. CCL2 is a potent inflammatory mediator and regulates collagen synthesis, and TGF-β1 is one of the major cytokines implicated in SSc by regulating extracellular matrix (ECM) production, fibroblast proliferation, and differentiation [26].

We also observed that coculture of B cells with SScHDF promotes the expression of TIMP1 and MMP9, which are implicated in the remodeling of the ECM. TIMP1 and MMP9 are upregulated in the serum of SSc patients and were found to correlate with disease activity [18,27]. SScHDF cocultured with B cells also expressed higher levels of α-SMA, which is a marker of differentiation of fibroblasts into contractile myofibroblasts*.* Myofibroblasts are specialized fibroblasts that show features of smooth muscle cell differentiation. They also synthesize collagen and are a major source of TGF-β1 and TIMP-1 during the fibrotic process [28,29].

Because BAFF is a pivotal cytokine for B-cell activation and its serum level is increased in SSc patients and correlates with the extent of skin fibrosis, we subsequently assessed whether BAFF could amplify B cell-induced fibrosis. First, we showed that BAFF was not secreted by dermal fibroblasts, B cells, or in cocultures. We then added BAFF and anti-IgM antibodies, because *in vitro* stimulation of B cells with BAFF requires their activation with anti-IgM antibodies [17]. BAFF exacerbated the effect of B cells on collagen production by SScHDF. BAFF also acted as a potent inducer of TIMP-1, α-SMA, CCL2, and IL-6. A similar induction of IL-6 by BAFF was also recently reported in monocytes of patients with primary Sjögren syndrome [30].

With transwell experiments, we also showed that cytokine release and collagen production are inhibited in the absence of cell-cell contact, indicating that the effect of B cells on SScHDF is related to membrane-bound factor(s). Recently, the interaction α_4_β_1_ integrin/VCAM-1 (vascular cell adhesion molecule 1) was identified as responsible for the interaction of blood B cells with skin fibroblasts [31]. By using blocking antibodies against integrin α_4_/VLA-4/CD49d, we determined that these molecules were not implicated in B cell-induced fibrosis in this model (data not shown). However, blocking TGF-β1 inhibited the effect of BAFF on collagen overproduction, suggesting that TGF-β1 is the key final mediator involved in B cell-induced fibrosis.

This suggests two hypotheses: (a) membrane TGF-β1 is directly implicated in the collaboration between B cells and fibroblasts; and (b) an as yet unknown cell-surface molecule on B cells (adhesion molecule, cytokine) induces TGF-β1 secretion by dermal fibroblasts.

These results reinforce the rationale for targeting B cells and/or BAFF in SSc. Recent open studies on limited populations of patients with SSc reported that B-cell depletion could result in decreased skin thickening [32-34]. This study suggests that BAFF could be an interesting therapeutic target in SSc, by its amplifying effect on profibrotic cytokines and collagen secretion. Interestingly, in the tight-skin mouse model, B-cell depletion or BAFF inhibition prevents skin fibrosis [35,36].

## Conclusion

B cells and BAFF play a pathogenic role in skin fibrosis in SSc by upregulating collagen secretion by dermal fibroblasts in a TGF-β1-dependent manner. Thus, these results demonstrate a new pathogenic role of B cells and BAFF in fibrosis and systemic sclerosis.

## Abbreviations

Alpha-SMA: α-smooth muscle actin; APRIL: A proliferation-inducing ligand; BAFF: B cell-activating factor; CCL2: Chemokine (C-C motif) ligand 2; COL1A1: Collagen type 1, α 1; ECM: Extracellular matrix; GAPDH: Glyceraldehyde 3-phosphate dehydrogenase; HDF: Human dermal fibroblasts; IL: Interleukin; MMP9: Matrix metallopeptidase 9; PBMC: Peripheral blood mononuclear cell; SSc: Systemic sclerosis; TGF-β1: Transforming growth factor beta 1; TIMP1: Tissue inhibitor of metalloproteinase 1; VCAM-1: Vascular cell-adhesion molecule 1; VLA-4: Very late antigen-4.

## Competing interests

The authors declare that they have no competing interests.

## Authors’ contributions

AF and GA carried out the experiments, and drafted and wrote the manuscript. JEG conceived of the study, participated in its design and coordination, performed the statistical analysis, and wrote the manuscript. EC, DW, JS, and SB participated in the design and coordination of the study and helped to draft the manuscript. All authors read and approved the final manuscript.

## Supplementary Material

Additional file 1: Figure S1 Fibroblasts increase B-cell survival *in vitro*. B cells alone or cocultured with fibroblasts were seeded in 24-well plates for 3 or 5 days. For transwell experiments, B cells (5 × 10^5^ cells) and HDF (10^5^ cells) were seeded in the upper and lower chambers, respectively. After 3 or 5 days, B-cell viability was determined by FACS analysis; vital B cells were brightly positive when stained with DiOC6 and excluded PI. Data are expressed as the median of duplicate samples of two independent experiments ± interquartile range. Click here for file

## References

[B1] Mouthon L SSc in 2011: from mechanisms to medicines Nat Rev Rheumatol 2012 15 72 74 22231235 10.1038/nrrheum.2011.203

[B2] Romano E Manetti M Guiducci S Ceccarelli C Allanore Y Matucci-Cerinic M The genetics of systemic sclerosis: an update Clin Exp Rheumatol 2011 15 S75 S86 21586222

[B3] LeRoy EC Medsger TA Jr Criteria for the classification of early systemic sclerosis J Rheumatol 2001 15 1573 1576 11469464

[B4] Novobrantseva TI Majeau GR Amatucci A Kogan S Brenner I Casola S Shlomchik MJ Koteliansky V Hochman PS Ibraghimov A Attenuated liver fibrosis in the absence of B cells J Clin Invest 2005 15 3072 3082 10.1172/JCI24798 16276416 PMC1265860

[B5] Komura K Yanaba K Horikawa M Ogawa F Fujimoto M Tedder TF Sato S CD19 regulates the development of bleomycin-induced pulmonary fibrosis in a mouse model Arthritis Rheum 2008 15 3574 3584 10.1002/art.23995 18975313

[B6] Kraaij MD van Laar JM The role of B cells in systemic sclerosis Biologics 2008 15 389 395 19707370 PMC2721390

[B7] Whitfield ML Finlay DR Murray JI Troyanskaya OG Chi JT Pergamenschikov A McCalmont TH Brown PO Botstein D Connolly MK Systemic and cell type-specific gene expression patterns in scleroderma skin Proc Natl Acad Sci USA 2003 15 12319 12324 10.1073/pnas.1635114100 14530402 PMC218756

[B8] Duncan MR Berman B Stimulation of collagen and glycosaminoglycan production in cultured human adult dermal fibroblasts by recombinant human interleukin 6 J Invest Dermatol 1991 15 686 692 1940439 10.1111/1523-1747.ep12483971

[B9] Sato S Hasegawa M Takehara K Serum levels of interleukin-6 and interleukin-10 correlate with total skin thickness score in patients with systemic sclerosis J Dermatol Sci 2001 15 140 146 10.1016/S0923-1811(01)00128-1 11532378

[B10] Saito F Tasaka S Inoue K Miyamoto K Nakano Y Ogawa Y Yamada W Shiraishi Y Hasegawa N Fujishima S Takano H Ishizaka A Role of interleukin-6 in bleomycin-induced lung inflammatory changes in mice Am J Respir Cell Mol Biol 2008 15 566 571 10.1165/rcmb.2007-0299OC 18096870

[B11] Verrecchia F Laboureau J Verola O Roos N Porcher R Bruneval P Ertault M Tiev K Michel L Mauviel A Farge D Skin involvement in scleroderma: where histological and clinical scores meet Rheumatology (Oxford) 2007 15 833 841 10.1093/rheumatology/kel451 17255134

[B12] Mackay F Schneider P Cracking the BAFF code Nat Rev Immunol 2009 15 491 502 10.1038/nri2572 19521398

[B13] Kalled SL The role of BAFF in immune function and implications for autoimmunity Immunol Rev 2005 15 43 54 10.1111/j.0105-2896.2005.00219.x 15790349

[B14] Mackay F Woodcock SA Lawton P Ambrose C Baetscher M Schneider P Tschopp J Browning JL Mice transgenic for BAFF develop lymphocytic disorders along with autoimmune manifestations J Exp Med 1999 15 1697 1710 10.1084/jem.190.11.1697 10587360 PMC2195729

[B15] Matsushita T Fujimoto M Hasegawa M Tanaka C Kumada S Ogawa F Takehara K Sato S Elevated serum APRIL levels in patients with systemic sclerosis: distinct profiles of systemic sclerosis categorized by APRIL and BAFF J Rheumatol 2007 15 2056 2062 17896803

[B16] Matsushita T Hasegawa M Yanaba K Kodera M Takehara K Sato S Elevated serum BAFF levels in patients with systemic sclerosis: enhanced BAFF signaling in systemic sclerosis B lymphocytes Arthritis Rheum 2006 15 192 201 10.1002/art.21526 16385515

[B17] Ng LG Sutherland AP Newton R Qian F Cachero TG Scott ML Thompson JS Wheway J Chtanova T Groom J Sutton IJ Xin C Tangye SG Kalled SL Mackay F Mackay CR B cell-activating factor belonging to the TNF family (BAFF)-R is the principal BAFF receptor facilitating BAFF costimulation of circulating T and B cells J Immunol 2004 15 807 817 15240667 10.4049/jimmunol.173.2.807

[B18] Kim WU Min SY Cho ML Hong KH Shin YJ Park SH Cho CS Elevated matrix metalloproteinase-9 in patients with systemic sclerosis Arthritis Res Ther 2005 15 R71 R79 10.1186/ar1454 15642145 PMC1064883

[B19] Henault J Robitaille G Senecal JL Raymond Y DNA topoisomerase I binding to fibroblasts induces monocyte adhesion and activation in the presence of anti-topoisomerase I autoantibodies from systemic sclerosis patients Arthritis Rheum 2006 15 963 973 10.1002/art.21646 16508979

[B20] Sato S Fujimoto M Hasegawa M Takehara K Tedder TF Altered B lymphocyte function induces systemic autoimmunity in systemic sclerosis Mol Immunol 2004 15 1123 1133 10.1016/j.molimm.2004.06.025 15482848

[B21] Sato S Hasegawa M Fujimoto M Tedder TF Takehara K Quantitative genetic variation in CD19 expression correlates with autoimmunity J Immunol 2000 15 6635 6643 11086109 10.4049/jimmunol.165.11.6635

[B22] Sato S Fujimoto M Hasegawa M Takehara K Altered blood B lymphocyte homeostasis in systemic sclerosis: expanded naive B cells and diminished but activated memory B cells Arthritis Rheum 2004 15 1918 1927 10.1002/art.20274 15188368

[B23] Chizzolini C Parel Y De Luca C Tyndall A Akesson A Scheja A Dayer JM Systemic sclerosis Th2 cells inhibit collagen production by dermal fibroblasts via membrane-associated tumor necrosis factor alpha Arthritis Rheum 2003 15 2593 2604 10.1002/art.11129 13130479

[B24] Chizzolini C Rezzonico R Ribbens C Burger D Wollheim FA Dayer JM Inhibition of type I collagen production by dermal fibroblasts upon contact with activated T cells: different sensitivity to inhibition between systemic sclerosis and control fibroblasts Arthritis Rheum 1998 15 2039 2047 10.1002/1529-0131(199811)41:11<2039::AID-ART20>3.0.CO;2-1 9811060

[B25] Kondo K Okada T Matsui T Kato S Date K Yoshihara M Nagata Y Takagi H Yoneda M Sugie I Establishment and characterization of a human B cell line from the lung tissue of a patient with scleroderma; extraordinary high level of IL-6 secretion by stimulated fibroblasts Cytokine 2001 15 220 226 10.1006/cyto.2000.0822 11237429

[B26] Abraham DJ Krieg T Distler J Distler O Overview of pathogenesis of systemic sclerosis Rheumatology (Oxford) 2009 15 iii3 iii7 10.1093/rheumatology/ken481 19487220

[B27] Young-Min SA Beeton C Laughton R Plumpton T Bartram S Murphy G Black C Cawston TE Serum TIMP-1, TIMP-2, and MMP-1 in patients with systemic sclerosis, primary Raynaud’s phenomenon, and in normal controls Ann Rheum Dis 2001 15 846 851 11502611 PMC1753839

[B28] Varga J Abraham D Systemic sclerosis: a prototypic multisystem fibrotic disorder J Clin Invest 2007 15 557 567 10.1172/JCI31139 17332883 PMC1804347

[B29] Kirk TZ Mark ME Chua CC Chua BH Mayes MD Myofibroblasts from scleroderma skin synthesize elevated levels of collagen and tissue inhibitor of metalloproteinase (TIMP-1) with two forms of TIMP-1 J Biol Chem 1995 15 3423 3428 10.1074/jbc.270.7.3423 7852429

[B30] Yoshimoto K Tanaka M Kojima M Setoyama Y Kameda H Suzuki K Tsuzaka K Ogawa Y Tsubota K Abe T Takeuchi T Regulatory mechanisms for the production of BAFF and IL-6 are impaired in monocytes of patients of primary Sjogren’s syndrome Arthritis Res Ther 2011 15 R170 10.1186/ar3493 22018243 PMC3308105

[B31] Couture P Paradis-Massie J Oualha N Thibault G Adhesion and transcellular migration of neutrophils and B lymphocytes on fibroblasts Exp Cell Res 2009 15 2192 2206 10.1016/j.yexcr.2009.04.013 19394331

[B32] Bosello S De Santis M Lama G Spano C Angelucci C Tolusso B Sica G Ferraccioli G B cell depletion in diffuse progressive systemic sclerosis: safety, skin score modification and IL-6 modulation in an up to thirty-six months follow-up open-label trial Arthritis Res Ther 2010 15 R54 10.1186/ar2965 20338043 PMC2888203

[B33] Daoussis D Liossis SN Tsamandas AC Kalogeropoulou C Kazantzi A Korfiatis P Yiannopoulos G Andonopoulos AP Is there a role for B-cell depletion as therapy for scleroderma? A case report and review of the literature Semin Arthritis Rheum 2010 15 127 136 10.1016/j.semarthrit.2009.09.003 20004954

[B34] Daoussis D Liossis SN Tsamandas AC Kalogeropoulou C Paliogianni F Sirinian C Yiannopoulos G Andonopoulos AP Effect of long-term treatment with rituximab on pulmonary function and skin fibrosis in patients with diffuse systemic sclerosis Clin Exp Rheumatol 2011 15 S17 S22 22244622

[B35] Hasegawa M Hamaguchi Y Yanaba K Bouaziz JD Uchida J Fujimoto M Matsushita T Matsushita Y Horikawa M Komura K Takehara K Sato S Tedder TF B-lymphocyte depletion reduces skin fibrosis and autoimmunity in the tight-skin mouse model for systemic sclerosis Am J Pathol 2006 15 954 966 10.2353/ajpath.2006.060205 16936269 PMC1698806

[B36] Matsushita T Fujimoto M Hasegawa M Matsushita Y Komura K Ogawa F Watanabe R Takehara K Sato S BAFF antagonist attenuates the development of skin fibrosis in tight-skin mice J Invest Dermatol 2007 15 2772 2780 17581616 10.1038/sj.jid.5700919

