# Supplementary figures and images for: B lymphocytes and B-cell activating factor promote collagen and profibrotic markers expression by dermal fibroblasts in systemic sclerosis
Source: Arthritis Res Ther. 2013 Oct 28;15(5):R168. doi: 10.1186/ar4352 (PMC3978899; doi:10.1186/ar4352)

# Supplementary figure 1

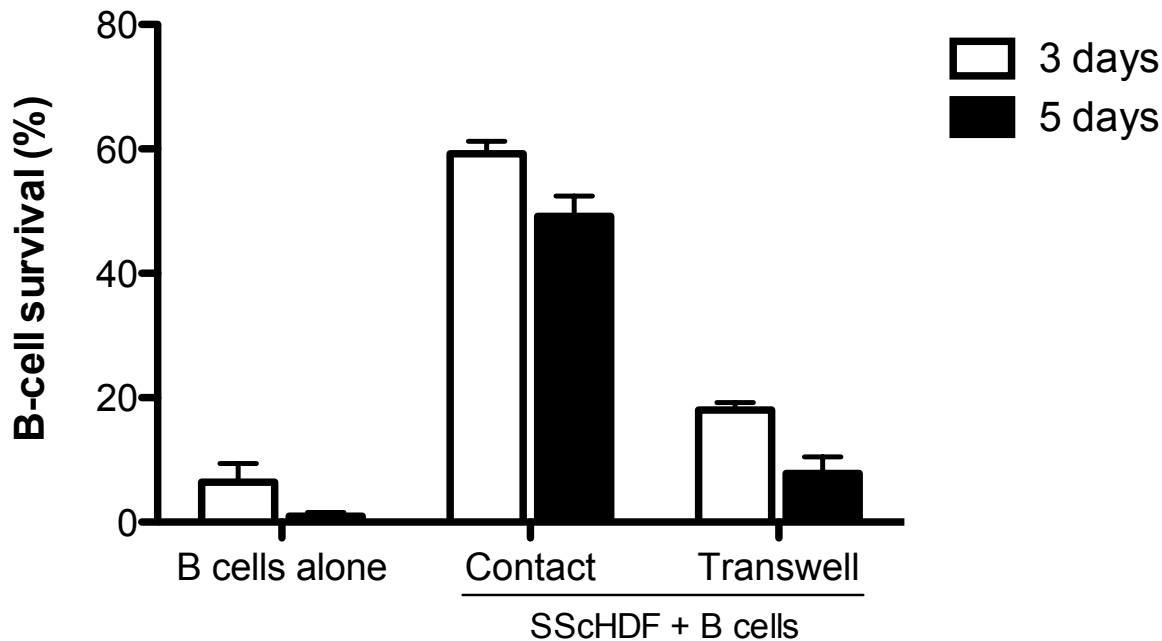

Supplement: Additional file 1: Figure S1 — Fibroblasts increase B-cell survival in vitro. B cells alone or cocultured with fibroblasts were seeded in 24-well plates for 3 or 5 days. For transwell experiments, B cells (5 × 10^5^ cells) and HDF (105 cells) were seeded in the upper and lower chambers, respectively. After 3 or 5 days, B-cell viability was determined by FACS analysis; vital B cells were brightly positive when stained with DiOC6 and excluded PI. Data are expressed as the median of duplicate samples of two independent experiments ± interquartile range. [file ar4352-S1.pdf]
